# Palliative Gastrointestinal Surgery in Patients With Advanced Peritoneal Carcinomatosis: Clinical Experience and Development of a Predictive Model for Surgical Outcomes

**DOI:** 10.3389/fonc.2021.811743

**Published:** 2022-01-13

**Authors:** Jolene Si Min Wong, Sze Min Lek, Daniel Yan Zheng Lim, Claramae Shulyn Chia, Grace Hwei Ching Tan, Chin-Ann Johnny Ong, Melissa Ching Ching Teo

**Affiliations:** ^1^ Department of Sarcoma, Peritoneal and Rare Tumours (SPRinT), Division of Surgery and Surgical Oncology, National Cancer Centre Singapore, Singapore, Singapore; ^2^ Department of Sarcoma, Peritoneal and Rare Tumours (SPRinT), Division of Surgery and Surgical Oncology, Singapore General Hospital, Singapore, Singapore; ^3^ SingHealth Duke-NUS Surgery Academic Clinical Program, Duke-NUS Medical School, Singapore, Singapore; ^4^ SingHealth Duke-NUS Oncology Academic Clinical Program, Duke-NUS Medical School, Singapore, Singapore; ^5^ Department of Anaesthesia and Surgical Intensive Care, Changi General Hospital, Singapore, Singapore; ^6^ Health Services Research Unit, Medical Board, Singapore General Hospital, Singapore, Singapore; ^7^ Laboratory of Applied Human Genetics, Division of Medical Sciences, National Cancer Centre Singapore, Singapore, Singapore; ^8^ Institute of Molecular and Cell Biology, ASTAR Research Entities, Singapore, Singapore

**Keywords:** advanced cancer, intestinal obstruction, palliation, palliative surgery, peritoneal carcinomatosis

## Abstract

**Background:**

Palliative gastrointestinal (GI) surgery potentially relieves distressing symptoms arising from intestinal obstruction (IO) in patients with advanced peritoneal carcinomatosis (PC). As surgery is associated with significant morbidity risks in advanced cancer patients, it is important for surgeons to select patients who can benefit the most from this approach. Hence, we aim to determine predictors of morbidity and mortality after palliative surgery in patients with PC. In addition, we evaluate the utility of the UC Davis Cancer Care nomogram (UCDCCn) and develop a simplified model to predict short-term surgical mortality in these patients.

**Methods:**

A retrospective review of patients with IO secondary to PC undergoing palliative GI surgery was performed. Logistic regression was used to determine independent predictors of 30-day morbidity and mortality after surgery. UCDCCn was evaluated using the area under the curve (AUC) for discriminatory power and the Hosmer-Lemeshow test for calibration. Our simplified model was developed using logistic regression and evaluated using cross-validation.

**Results:**

A total of 254 palliative GI surgeries were performed over a 10-year duration. The 30-day morbidity and mortality were 43% (n = 110) and 21% (n = 53), respectively. Preoperative albumin, age, and emergency nature of surgery were significant independent predictors for 30-day morbidity. A simplified model using preoperative Eastern Cooperative Oncology Group (ECOG) status and albumin (AUC = 0.71) achieved better predictive power than UCDCCn (AUC = 0.66) for 30-day mortality.

**Conclusion:**

Good ECOG status and high preoperative albumin levels were independently associated with good short-term outcomes after palliative GI surgery. Our simplified model may be used to conveniently and efficiently select patients who stand to benefit the most from surgery.

## 1 Introduction

Peritoneal carcinomatosis (PC) is an end-stage presentation of up to 50% of advanced cancer patients with various primary tumors (1, 2). Debilitating gastrointestinal (GI) symptoms due to complex, multilevel intestinal obstruction (IO) are common and may not be adequately palliated with medical or endoscopic therapy alone (3). As such, though infrequently publicized, palliative surgeries make up approximately one-fifth of all surgical procedures performed at any major cancer center annually (4). In fact, most report high rates of clinical success ranging from 80% to 100% after palliative GI surgery for PC-associated IO (5, 6).

Though a direct and effective means of palliation in IO, surgery is associated with significant morbidity risks. A systematic review of 17 retrospective studies on surgical management of malignant bowel obstruction found that serious complications occurred in up to 44% of patients, and mortality rates ranged from 6% to 32% (5). Citing high morbidities and in-hospital deaths among advanced cancer patients undergoing palliative surgery, some physicians adopt a blanket “no surgery” approach in favor of medical treatment alone among palliative PC patients (7, 8). This misconception deprives suitable surgical candidates of a treatment modality that can provide good palliation during end of life. As such, there is a need to identify palliative PC patients who will benefit the most from surgery while adopting discretion when offering a surgical mode of palliation in those in whom poor outcomes are expected.

The UC Davis nomogram was developed to predict 30-day morbidity and mortality among patients with disseminated malignancy who had undergone surgical intervention (9). With the use of data from the American College of Surgeons National Surgical Quality Improvement Program (ACS NSQIP), preoperative factors affecting an individual’s risk of perioperative morbidity and mortality were identified. Thirteen and fourteen factors including “do not resuscitate (DNR)” status, age, weight loss >10%, dyspnea, functional dependence, ascites, chronic steroid use, active sepsis, serum creatinine level, serum albumin level, serum white blood cell (WBC), serum hematocrit and acuity of surgical procedure, and procedure type were found to be independently associated with postoperative complications and death, respectively. With the use of the above factors, nomograms predictive of the probability of experiencing a postoperative event were then constructed. While comprehensive, the model has not been independently validated and may be cumbersome when applied in clinical practice due to its complexity.

As such, our study aims to report our clinical experience in palliative GI surgery in the context of PC and evaluate the utility of the UC Davis model in predicting perioperative outcomes in our patient cohort. We also aim to develop a simplified model to predict 30-day morbidity and mortality among patients undergoing palliative surgery.

## 2 Materials and Methods

A retrospective review of PC patients who underwent palliative surgery for IO at the Singapore General Hospital was performed from January 2009 to January 2019. Patients with PC from a variety of primary malignancies, including GI, gynecological, hepato-pancreatico-biliary, and others, were included. Patient demographics, perioperative variables, tumor characteristics, and postoperative morbidity and mortality outcomes were obtained from medical records.

The study was conducted with the approval of the ethics board.

### 2.1 Definitions

#### 2.1.1 Peritoneal Carcinomatosis and Intestinal Obstruction

All patients had a histologically proven diagnosis of malignancy and histologically or radiologically proven metastases, specifically metastases to the peritoneum, at the time of surgery. IO was defined clinically based on signs and symptoms of obstruction such as abdominal distention, abdominal pain, nausea and vomiting, constipation, inability to pass air, or radiologically on imaging modalities performed (10).

#### 2.1.2 Preoperative, Intraoperative, and Postoperative Variables

The following comorbid conditions were determined to be absent or present based on ACS NSQIP criteria (11). Dyspnea was defined as the presence of labored breathing on exertion or at rest. Significant weight loss was defined as weight loss of 10% in the previous 6 months. Preoperative sepsis was defined as a positive bacterial culture identified in addition to two or more of the following criteria: fever, tachycardia, tachypnea, leukocytosis, and anion gap acidosis. Preoperative chemotherapy or radiotherapy was defined as the administration of chemotherapy within 30 days and radiotherapy within 90 days before surgery.

Intraoperatively, the type of surgical procedures was stratified to consider if GI resection, multi-visceral resection, and other abdominal surgical procedures such as adhesiolysis were performed as per the ACS NSQIP classification. We further collected information on the type of bowel resection, anastomoses, and stoma fashioned. Emergency cases were designated by the primary surgeon after considering the clinical circumstances surrounding palliative surgical interventions.

Data on postoperative complications were collected and included organ-specific complications (hematological, cardiac, respiratory, neurologic, abdominal, and others). This was in line with ACS NSQIP-reported complication codes. Unplanned readmissions and Calvien–Dindo-based classification of major and minor postoperative complications were recorded as well (12).

#### 2.1.3 30-Day Overall Morbidity and Mortality

Morbidity and mortality were considered at 30 days calculated from the date of palliative surgery.

### 2.2 Statistical Analysis

The baseline statistics of the cohort were summarized with a mean (SD) for continuous variables and N (%) for categorical variables. Univariate statistical testing was performed for significant associations between individual preoperative and postoperative variables, with 30-day mortality and 30-day morbidity. We used t-test for continuous variables and chi-square testing for categorical variables.

For the development of the multivariate and simplified multivariate models, the data were split into training and test sets in a 7:3 ratio. Continuous variables were scaled and normalized. Multivariate logistic regression was performed on the training set, with preoperative variables used as predictive factors. The backward method of multivariate logistic regression was used in view of the large number of potential predictors identified. To evaluate discriminative power, the area under the curve (AUC) was evaluated on the test set, with the confidence limits determined by bootstrapping. Sensitivity and specificity were calculated using Youden’s method to determine the optimal cutoff point.

UC Davis 30-day morbidity and mortality predicted probabilities were calculated from the UC Davis Nomogram. The AUC was used to determine its discriminatory power and the Hosmer–Lemeshow test (H-L test) for calibration. 95% CIs for the AUC were determined *via* bootstrapping.

Statistical analysis was performed using the Statistical Package for Social Sciences version 24 (SPSS Inc., Chicago, IL, USA), R version 3.6.1, and Python 3.7. Statistical significance was defined at the 0.05 level.

## 3 Results

### 3.1 Baseline Characteristics

A total of 254 palliative GI surgeries were performed among PC patients over a 10-year duration. The median age of our patients was 61.5 (range 52–71). The most common site of primary malignancy was the colon (42.6%). All patients had radiographic or grossly seen peritoneal disease, which was subsequently confirmed on histopathologic specimens; 24.4% had lung metastases, and 31.8% had liver metastases in addition to peritoneal metastases. The demographic and clinical characteristics of the patients are presented in **Table 1**.

**Table 1:** Demographics and clinical characteristics of palliative GI surgery patients.

Variable	Mean or N (%)
Age	61.5 (52.3–71)
Male	101 (40%)
Smoker	10 (4%)
**Site of primary malignancy**	
Lung	7 (3%)
Stomach	32 (13%)
Pancreas	14 (6%)
Colon	108 (43%)
Ovary	33 (13%)
Endometrial	6 (2%)
Cervix	5 (2%)
Others	49 (19%)
Presence of lung metastases	62 (24%)
Presence of liver metastases	81 (32%)
**Comorbid disease**	
Hypertension (requiring medication)	89 (35%)
Diabetes (requiring medication)	49 (19%)
Chronic obstructive pulmonary disease	3 (1%)
Myocardial infarction	5 (2%)
Congestive heart failure	4 (2%)
Peripheral vascular disease	1 (0%)
Renal failure	7 (3%)
Dialysis	4 (2%)
**Preoperative clinical characteristics**	
Emergency surgery	172 (68%)
Prehospital location (home)	249 (98%)
Independent functional status	246 (97%)
DNR status	5 (2%)
Chemotherapy use (within 30 days)	51 (20%)
Radiotherapy use (within 90 days)	4 (2%)
Weight loss > 10% within 6 months	79 (31%)
Steroid use	17 (7%)
Ascites	161 (63%)
Bleeding disorder	4 (2%)
Dyspnea at rest	8 (3%)
Impaired sensorium	5 (2%)
Pneumonia	5 (2%)
Sepsis	30 (12%)
ECOG status	
0	2 (1%)
1	141 (56%)
2	100 (39%)
3	11 (4%)
Hematocrit (%) (median, IQR)	33.9 (30.9–37.5)
WBC (×10^9^/L) (median, IQR)	8.3 (6.2–11.4)
Albumin (g/L) (median, IQR)	31 (27–35)
Creatinine (μmol/L) (median, IQR)	58 (47–81)
**Procedure type**	
Gastrointestinal resection	219 (86%)
Multi-visceral resection	22 (9%)
Lysis of adhesions	7 (3%)
**Anastomoses**	
Any anastomosis	164 (65%)
Gastro-jejunal	17 (7%)
Small bowel–small bowel	63 (24%)
Small bowel–large bowel	86 (34%)
Large bowel–large bowel	13 (5%)
**Stoma**	
Any stoma	91 (35%)
Gastrostomy	4 (2%)
Jejunostomy	4 (2%)
Ileostomy	35 (14%)
Colostomy	46 (18%)

GI, gastrointestinal; DNR, do not resuscitate; ECOG, Eastern Cooperative Oncology Group; IQR, interquartile range; WBC, white blood cell.

### 3.2 Predictors of 30-Day Morbidity and Mortality

The 30-day morbidity after palliative GI surgery was 43% (n = 110). The most common complications included hematologic complications 31.1% (i.e., requiring multiple blood product transfusions), intra-abdominal complications 29.5% (i.e., intra-abdominal sepsis and collections), respiratory complications 22.0%, wound 18.1%, and cardiac 14.1% complications. Minor (Calvien–Dindo grades 1 and 2) and major (Grade 3 onwards) complications occurred in 43% and 57% of patients, respectively. Of the patients, 20.1% and 5.9% had unplanned readmissions and unplanned reoperations, respectively.

**Figure 1:**
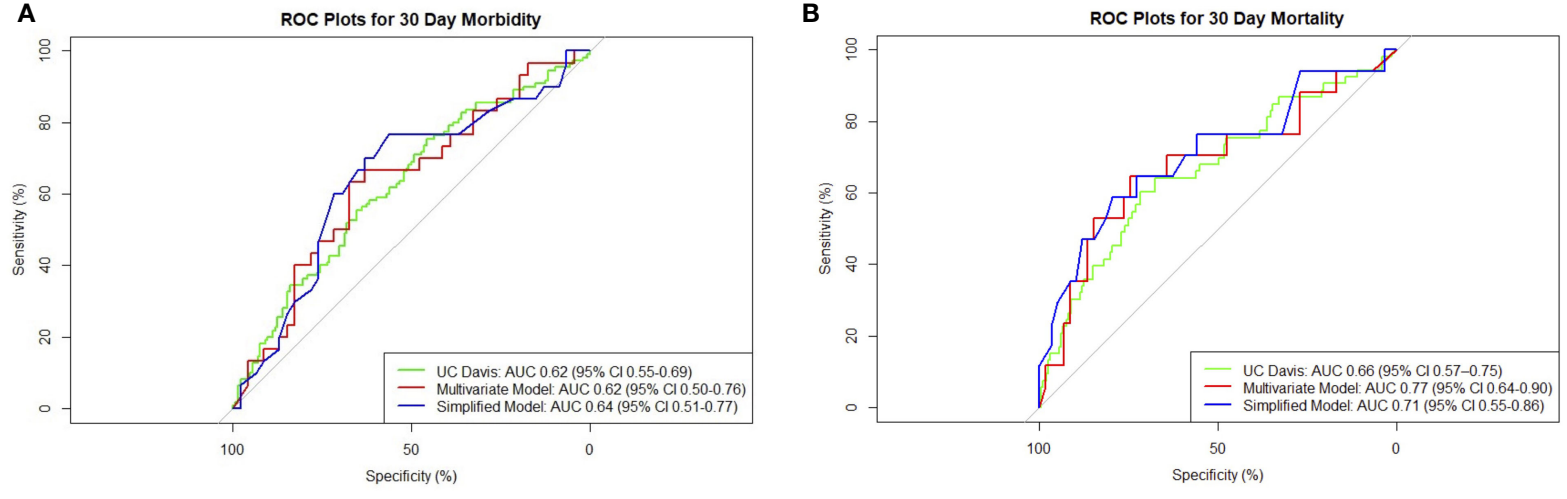
Composite receiver operating characteristic (ROC) plots for 30-day **(A)** morbidity and **(B)** mortality models.

Low preoperative albumin and hematocrit, dyspnea, preoperative use of steroids, and preoperative sepsis were predictors of 30-day morbidity on univariate analysis (**Table 2**). On multivariate analysis, preoperative albumin, age, and emergency nature of surgery were found to be independent significant predictors with an AUC of 0.62 (95% CI 0.50–0.76, **Figure 1**).

**Table 2:** Predictors of 30-day morbidity and mortality after palliative GI surgery.

Variable	30-day morbidity (n = 110)	p-Value	30-day mortality (n = 53)	p-Value
Age	64 (56–71)	0.09	63 (57–69)	0.27
Male	42	0.70	16	0.11
Smoker	6	0.34	1	0.69
**Site of primary malignancy**				
Lung	3	1	1	1
Stomach	15	0.70	7	0.81
Pancreas	8	0.41	5	0.17
Colon	45	0.70	24	0.64
Ovary	16	0.57	1	**0.004**
Endometrial	4	0.41	3	0.1
Cervix	1	0.39	0	0.58
Others	1	0.43	12	NA
Presence of lung metastases	29	0.56	14	0.72
Presence of liver metastases	35	1	20	0.32
**Comorbid disease**				
Hypertension	40	0.79	53	0.12
	22	0.74	18	1
Diabetes	0	0.26	12	0.43
Chronic obstructive pulmonary disease	2	1	0	1
Myocardial infarction	3	0.32	1	1
Congestive heart failure	0	1	0	0.58
Peripheral vascular disease	5	0.24	0	1
Renal failure	2	1	1	1
Dialysis			0	0.58
**Preoperative clinical characteristics**				
Emergency Surgery	69	0.18	38	0.51
Prehospital Location (home)	109	0.39	51	0.27
Independent functional status	108	0.47	50	0.36
DNR status	1	0.39	1	1
Chemotherapy use (within 30 days)	24	0.64	9	0.69
Radiotherapy use (within 90 days)	1	0.64	1	1
Weight loss > 10% within 6 months	33	0.79	17	0.86
Steroid use	13	**0.005**	3	1
Ascites	74	0.29	40	**0.05**
Bleeding disorder	3	0.32	0	0.58
Dyspnea at rest	8	**0.001**	2	0.67
Impaired sensorium	3	0.65	0	0.58
Pneumonia	2	1	2	0.27
Sepsis	23	**<0.001**	4	0.34
ECOG status		0.13		**0.03**
0	0		0	
1	54		22	
2	51		26	
3	5		5	
Hematocrit (%) (median, IQR)	32.7 (29.7–36.1)	**0.003**	33.8 (30.0–37.1)	0.23
WBC (×10^9^/L) (median, IQR)	8.8 (6.5–12.1)	0.14	8.8 (6.3–12.1)	0.2
Albumin (g/L) (median, IQR)	29.5 (26–34)	**0.003**	28 (24–32)	**<0.001**
Creatinine (μmol/L) (median, IQR)	56 (43–76)	0.58	55 (45–73)	0.94
**Procedure type**				
Gastrointestinal resection	96	0.85	49	**0.02**
Multi-visceral resection	10		0	NA
Lysis of adhesions	2		2	
**Anastomoses**				
Any anastomosis	66	0.19	29	0.1
Gastro-jejunal	6	0.61	5	0.36
Small bowel–small bowel	30	0.46	10	0.28
Small bowel–large bowel	33	0.29	17	0.87
Large bowel–large bowel	6	1	2	1
**Stoma**				
Any stoma	44	0.24	23	0.2
Gastrostomy	3	0.32	2	0.19
Jejunostomy	4	0.03	2	0.19
Ileostomy	19	0.20	5	0.37
Colostomy	19	0.87	11	0.55

GI, gastrointestinal; DNR, do not resuscitate; ECOG, Eastern Cooperative Oncology Group; IQR, interquartile range; WBC, white blood cell; NA, Not applicable. Values in bold indicate p < 0.05.

The 30-day mortality was 21% (n = 53). As all patients had advanced cancer with a prognosis of less than 1 year, it was found that 81% (n = 206) demised within 1 year of palliative surgical intervention. Median survival was 109 days (range 43–265).

The presence of ascites, high Eastern Cooperative Oncology Group (ECOG) status, low albumin, and extent of surgery were associated with higher 30-day mortality on univariate analysis (**Table 2**). Patients with ovarian primaries had a significantly lowered risk of death at 30 days (p = 0.004). On multivariate logistic regression analysis with backward variable selection, only ECOG status and preoperative albumin levels were found to be independent significant predictors (**Table 3**). The final multivariable model had an AUC of 0.77 (95% CI 0.64–0.90, **Figure 1**) and sensitivity and specificity of 0.76 and 0.75, respectively.

**Table 3:** Model summary of multivariable model for 30-day mortality.

Variable	Adjusted odds ratio (95% CI)	p-Value
**Site of primary malignancy**		
Ovary	0.20 (0.01–1.07)	0.13
Endometrial	0 (0–999)	0.99
**Preoperative clinical characteristics**		
DNR status	0 (0–999)	0.99
Impaired sensorium	0 (0–999)	0.99
Sepsis	0.20 (0.02–1.03)	0.10
ECOG status	1.59 (1.07–2.38)	**0.023**
WBC	1.50 (0.98–2.33)	0.06
Albumin	0.60 (0.39–0.89)	**0.016**

DNR, do not resuscitate; ECOG, Eastern Cooperative Oncology Group; WBC, white blood cell. Values in bold indicate p < 0.05.

### 3.3 Utility of the UC Davis Nomogram in Our Patient Cohort

**Figure 2:**
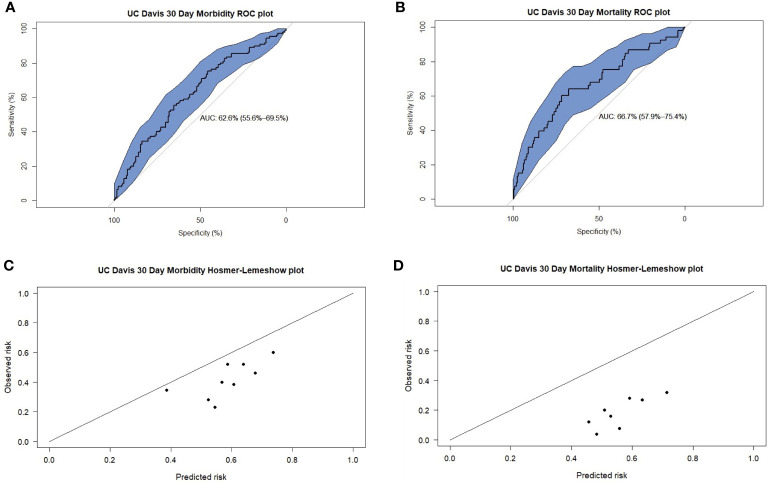
Receiver operating characteristic (ROC) plots for UC Davis predictions of 30-day **(A)** morbidity and **(B)** mortality. Hosmer-Lemeshow (H-L) plots for UC Davis predictions of 30-day **(C)** morbidity and **(D)** mortality.

In the prediction of 30-day morbidity, the UC Davis Nomogram had an AUC of 0.62 (95% CI 0.55–0.69), and the H-L test had a p-value of 0.99. This indicated poor discriminative power but acceptable calibration (**Figure 2**).

In the prediction of 30-day mortality, the UC Davis Nomogram had an AUC of 0.66 (95% CI 0.57–0.75) for 30-day mortality, indicating poor discriminative power. The H-L test had a p-value <0.05, indicating poor calibration.

### 3.4 Simplified Model for 30-Day Morbidity and Mortality Outcomes for Palliative Gastrointestinal Surgery Patients

To develop a simplified model, we entered the significant variables of ECOG and preoperative albumin found on multivariable regression into a new logistic model. For 30-day morbidity, the simplified model had an AUC of 0.64 (95% CI 0.51–0.77) and sensitivity and specificity of 0.77 and 0.57, respectively. For 30-day mortality, the simplified model had an AUC of 0.71 (95% CI 0.55–0.86) and sensitivity and specificity of 0.59 and 0.80, respectively. The receiver operating characteristic (ROC) plot summaries comparing UC Davis, multivariate, and simplified models for morbidity and mortality are plotted in **Figure 1**. The model summaries are included in **Table 4**.

**Table 4:** Model summary of simplified models.

Variable	Adjusted odds ratio (95% CI)	p-Value
**30-Day morbidity**		
ECOG status	1.26 (0.94–1.71)	0.12
Albumin	0.71 (0.52–0.97)	**0.03**
**30-Day mortality**		
ECOG status	1.56 (1.08–2.26)	**0.018**
Albumin	0.60 (0.40–0.89)	**0.012**

ECOG, Eastern Cooperative Oncology Group. Values in bold indicate p < 0.05.

**Figure 3:**
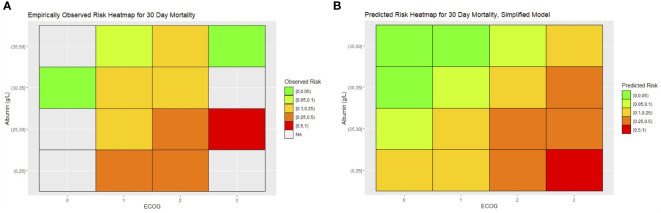
**(A)** Empirically observed and **(B)** predicted risk heatmaps for 30-day mortality.

To translate the 30-day mortality simplified model into a clinical tool for prediction, we constructed heatmaps of expected risk. The heatmap skeleton was a matrix with ECOG on one axis and preoperative albumin on the other. Preoperative albumin was stratified by rounding off the observed quartile of albumin in the cohort to the nearest 5 g/L. The average expected risk was determined for each cell of the heatmap and colored with a gradient of green to red, with green representing the lowest risk. We constructed a similar heatmap with the empirically observed mortality in our cohort, with the observed mortality aggregated and color-coded for each cell (**Figure 3**).

## 4 Discussion

PC complicated with IO is one of the most common indications for surgical intervention among advanced cancer patients (10). The peritoneum houses intra-abdominal organs and is inevitably involved in disseminated end-stage cancer. Hence, PC represents a “common end point” of most advanced cancers, where patients may develop complex, multilevel IO and thus suffer from progressive inability to tolerate food, intractable abdominal pain, and distension (13, 14). Surgery usually entails bowel resection, bypass, and/or creation of a decompressive ostomy (15). Among our patients, a majority (86%) required GI resection with a frequent need for bowel anastomoses and stoma creation. As such, palliative GI surgery when performed in PC patients represents a unique group where surgery may be extensive and associated with significant postoperative complications (16).

In one of the largest series of palliative surgical procedures, Miner et al. observed at 30-day postoperative morbidity of 29% and mortality of 11% (6). In the context of PC, a systemic review of 17 retrospective studies including 868 patients found that serious complications occurred in up to 44% of patients, while mortality ranged from 6% to 32% (5). Similarly, we found that 30-day morbidity and mortality were 43% and 21%, respectively, among PC patient who had undergone palliative GI surgery. However, while opponents of palliative surgery tend to focus on the high complications rates, many fail to acknowledge the high symptom resolution (up to 80% to 100%) and low “symptom recurrence” rates after surgical intervention. In fact, our study revealed that none of our patients required repeated operation intervention for IO. Defining the parameters of surgical success is thus of paramount importance—a patient’s morbidity or mortality shortly after palliative surgery should not constitute a failure if the wishes of the patients were fulfilled and they had enjoyed a “good” end-of-life experience from their perspective with adequate symptom resolution.

At the time of consideration of palliative surgery among advanced PC patients, the prognosis is often dismal, and predicted survival is less than 1 year. Therefore, it was not surprising that our reported median survival was 109 days and 80% had demised within 1 year after surgery. The importance of identifying factors predictive of short-term mortality is essential to select patients who will benefit the most from palliative surgical interventions. While the existing UC Davis nomogram is comprehensive and useful in predicting 30-day surgical morbidity and mortality, its direct application among PC patients undergoing extensive GI surgery is questionable. The UC Davis cohort is composed of patients who had gone through a variety of surgical procedures, such as vascular, skin and soft tissue, hepatobiliary, and GI interventions. This is distinct from a PC cohort, as the extent of gut manipulation and anastomoses often results in higher rates of perioperative morbidity and mortality. Therefore, when applied to our PC patients, AUC was found to be less than 0.7, representing poor discriminatory power for both 30-day mortality and morbidity outcomes.

Hence, there was a need to devise a risk model that was more applicable to PC patients undergoing palliative GI surgery. Our model comprising ECOG status and serum albumin was found to achieve superior predictive power over the UC Davis model. As such, we advocate the use of this simplified model and translated heatmap as quick clinical tools to aid operative risk discussion.

The impact of preoperative albumin levels on outcomes suggests a role for optimization through preoperative parenteral nutrition in selected PC patients planned for palliative GI surgery. While enteral nutrition has been found to be superior to total parenteral nutrition (TPN) in improving outcomes prior to surgery, this is often not possible in the PC cohort due to multilevel IO (17). In Crohn’s disease, characterized by gut failure, not dissimilar to PC patients, TPN given 60 days before major abdominal GI surgery resulted in reduced rates of postoperative complications (18). As such, it is possible that a trial of preoperative TPN can improve albumin levels and lead to improved outcomes among palliative PC patients who do not present with surgical emergencies.

A limitation of this analysis is its inability to account for patients who might have been eligible for palliative surgery but were not operated on because of other factors such as patient decisions or surgeon assessment. We note that our cohort had very few ECOG 0 or 3 patients. Patients may not have been operated on because they were either deemed good candidates for further conservative management (such as ECOG 0 patients, who may have had resolution of obstruction with further nonoperative management) or too poor candidates for surgical management (such as ECOG 3 patients). This can result in paradoxical results, which may be seen when the predicted and observed heatmaps are compared for 30-day mortality. In ECOG 3 patients, those with high albumin >35 g/L experienced lower than predicted risks, while no patients with moderate albumin levels of 25–30 g/L were operated on. Bias arising from surgeon selection of perceived good candidates for surgery may have caused this apparent paradox and may cause overly optimistic risk estimates (such as in the case of the UC Davis model). Further refinement of risk estimates may be possible if further data are collected on such marginal patients.

In addition, the authors believe that traditional factors used to evaluate surgical efficacy such as the abovementioned postoperative complications and survival fall short of measuring outcomes most meaningful to advanced cancer patients during end of life. Survival reported in quantitative terms without reporting its quality does not attest to overall patient experience and incentivizes surgeons and non-surgeons alike to adopt measures that prolong rather than improve life. Instead, future studies should evaluate measures of quality of life, functional independence, and freedom from symptoms after surgery, which are both clinically important and meaningful to advanced cancer patients undergoing palliative surgery. Therefore, the true “value” of palliative surgery should be considered based on the preferences, expectations, and goals of care of each patient nearing end of life (19).

In conclusion, we found that a good ECOG status and high preoperative albumin levels were independently associated with good short-term outcomes after palliative GI surgery. The UC Davis nomogram showed poor performance in our cohort for the prediction of both mortality and morbidity in our patient cohort. We propose that our simplified 30-day mortality risk model and heatmap may be used as a quick stratification tool for surgeons discussing potential operative risks with patients and that further research will be needed to develop a similar tool for 30-day morbidity.

## Data Availability Statement

The original contributions presented in the study are included in the article/supplementary material. Further inquiries can be directed to the corresponding author.

## Ethics Statement

The studies involving human participants were reviewed and approved by SingHealth Centralised Institutional Review Board. The patients/participants provided their written informed consent to participate in this study.

## Author Contributions

Conceptualization: MCCT. Methodology: MCCT, JSMW, and SML. Validation: JSMW, SML, DYZL, CSC, GHCT, and C-AJO. Formal analysis: JSMW, SML, DYZL, CSC, GHCT, and C-AJO. Investigation: JSMW, SML, DYZL, CSC, GHCT, and C-AJO. Resources: CSC, GHCT, C-AJO, and MCCT. Data curation: JSMW and SML. Writing—original draft: JSMW, SML, and DYZL. Writing—review and editing: JSMW, SML, DYZL, CSC, GHCT, C-AJO, and MCCT. Visualization: JSMW, SML, and DYZL. Supervision: CSC, GHCT, C-AJO, and MCCT. Project administration: CSC, GHCT, C-AJO, and MCCT. Funding acquisition: JSMW, CSC, C-AJO, and MCCT. All authors contributed to the article and approved the submitted version.

## Funding

This study is supported by the NCCS Cancer Fund (Research) and SingHealth Duke-NUS Academic Medicine Centre, facilitated by the Joint Office of Academic Medicine (JOAM). C-AJO is supported by the National Research Council Transition Award (NMRC/TA/0061/2017). All the funding sources had no role in the study design, data interpretation, or writing of the manuscript.

## Conflict of Interest

The authors declare that the research was conducted in the absence of any commercial or financial relationships that could be construed as a potential conflict of interest.

## Publisher’s Note

All claims expressed in this article are solely those of the authors and do not necessarily represent those of their affiliated organizations, or those of the publisher, the editors and the reviewers. Any product that may be evaluated in this article, or claim that may be made by its manufacturer, is not guaranteed or endorsed by the publisher.
